# Genome-wide association study for grain zinc concentration in bread wheat (*Triticum aestivum L.*)

**DOI:** 10.3389/fpls.2023.1169858

**Published:** 2023-04-03

**Authors:** Jianhui Ma, Miaomiao Ye, Qianqian Liu, Meng Yuan, Daijing Zhang, Chunxi Li, Qingdong Zeng, Jianhui Wu, Dejun Han, Lina Jiang

**Affiliations:** ^1^ College of Life Science, Henan Normal University, Xinxiang, China; ^2^ State Key Laboratory of Crop Stress Biology in Arid Areas, Northwest A&F University, Yangling, Shanxi, China

**Keywords:** *Triticum aestivum* L., grain Zn concentration, genome-wide association study, quantitative trait loci, candidate gene

## Abstract

**Introduction:**

Zinc (Zn) deficiency causes serious diseases in people who rely on cereals as their main food source. However, the grain zinc concentration (GZnC) in wheat is low. Biofortification is a sustainable strategy for reducing human Zn deficiency.

**Methods:**

In this study, we constructed a population of 382 wheat accessions and determined their GZnC in three field environments. Phenotype data was used for a genome-wide association study (GWAS) using a 660K single nucleotide polymorphism (SNP) array, and haplotype analysis identified an important candidate gene for GZnC.

**Results:**

We found that GZnC of the wheat accessions showed an increasing trend with their released years, indicating that the dominant allele of GZnC was not lost during the breeding process. Nine stable quantitative trait loci (QTLs) for GZnC were identified on chromosomes 3A, 4A, 5B, 6D, and 7A. And an important candidate gene for GZnC, namely, TraesCS6D01G234600, and GZnC between the haplotypes of this gene showed, significant difference (P ≤ 0.05) in three environments.

**Discussion:**

A novel QTL was first identified on chromosome 6D, this finding enriches our understanding of the genetic basis of GZnC in wheat. This study provides new insights into valuable markers and candidate genes for wheat biofortification to improve GZnC.

## 1 Introduction

Zinc (Zn) is an essential microelement for human normal metabolism. Zn deficiency causes serious diseases, such as liver cirrhosis, dwarfism, coronary heart disease, visual disorders, reproductive organ development disorders, and even cancer (Hambidge, 2000). Furthermore, approximately 17% of the world population is affected by Zn deficiency, especially women and children in developing countries (Shah and Sachdev, 2006; Wessells and Brown, 2012; Black et al., 2013). Bread wheat (*Triticum aestivum* L.) is one of the most important cereal crops in the world; its products provide approximately 20% of the energy and protein in the human diet (Ludwig and Slamet-Loedin, 2019). However, the grain Zn concentration (GZnC) is below the minimum level (38 mg/kg) required to meet human needs and cannot satisfy the demand from the people consuming wheat as their main staple food (Bouis et al., 2011). Therefore, increasing wheat GZnC is an important goal in wheat breeding.

Owing to the important role of wheat in human nutrition, wheat biofortification can be used as a cost-effective strategy to alleviate Zn deficiency, especially in low-income countries, where most people rely on cereal foods for a basic diet (Velu et al., 2014; Andersson et al., 2017; Ma et al., 2022). However, owing to the ambiguity of the genetic architecture and molecular processes regulating Zn homeostasis in wheat, breeding outstanding varieties with high GZnC is a difficult challenge (Gupta et al., 2021). Identification of molecular markers that are closely linked to QTLs controlling complex quantitative traits, such as grain iron, zinc, or protein concentration, are a goal on their own to facilitate the development of biofortified wheat cultivars through marker-assisted breeding, whereby, improving our understanding of the genetic basis of GZnC in wheat demands identifying as many causal loci as possible. Currently, genome-wide association study (GWAS) is the most popular method for analysing the genetic basis of complex traits in wheat (Breseghello and Sorrells, 2006; Bradbury et al., 2007).

Recently, some studies have been conducted to identify QTLs for wheat GZnC by GWAS to further improvement. Specifically, to the best of our knowledge, nine GWAS studies have been conducted to identify QTLs and marker-trait associations (MTAs) for wheat GZnC. Thus, for example, Velu et al. identified 39 significant MTAs for wheat GZnC located on chromosomes 1A, 2A, 2B, 2D, 5A, 6B, 6D, 7B, and 7D, explaining 5% to 10.5% of the phenotypic variation (Velu et al., 2018). In turn, Alomari et al. detected 40 MTAs on chromosomes 2A, 3A, 3B, 4A, 4D, 5A, 5B, 5D, 6D, 7A, 7B, and 7D for wheat GZnC, which explained 2.5% to 5.2% of the phenotypic variation (Alomari et al., 2018). Similarly, Cu et al. identified 72 MTAs for wheat GZnC on chromosomes 1A, 2A, 3A, 4A, 5B, and 7A, explaining 3.7% to 5.2% of the phenotypic variation (Cu et al., 2020). Furthermore, Liu et al. detected 16 QTLs for wheat GZnC on chromosomes 1B, 3A, 3D, 4A, 4B, 5A, 5D, 6B, 6D, and 7D, explaining 2.7% to 6.6% of the phenotypic variation (Liu et al., 2021). In turn, Zhou et al. detected 29 QTLs on chromosomes 1A, 1B, 1D, 2B, 2D, 3A, 3B, 3D, 4A, 4B, 5A, 5B, 6A, 6B, 6D, and 7A, which explained 9.75% to 24.77% of the phenotypic variation (Zhou et al., 2020). Tong et al. identified 25 QTLs on chromosomes 1A, 2A, 3A, 3B, 5A, 5D, 6A, 6B, 6D, 7A, 7B, and 7D, which explained 7.73% to 13.57% of the phenotypic variation (Tong et al., 2022). Rathan et al. identified two MTAs on chromosomes 1A and 7B, explaining 6.35% to 7.60% of the phenotypic variation (Rathan et al., 2022). Juliana et al. identified 67 MTAs on chromosomes 1A, 1B, 2A, 2D, 3B, 4A, 5A, 5B, 5D, 6B, 6D, and 7B, among which the maximum phenotypic variation explained is 7.3% (Juliana et al., 2022). Krishnappa et al. found five MTAs on chromosomes 2B, 5B, 6A, and 7B, contributing to 5.7% to 10.9% of the phenotypic variation (Krishnappa et al., 2022). However, they are insufficient for further map-based cloning.

Using of different germplasm resources and high resolution genotyping techniques may contribute to identify important QTLs for wheat GZnC; therefore enriching the genetic information. In this study, 382 wheat accessions were used to determine wheat GZnC in three environments to analysis the changes of wheat GZnC. Additionally, a GWAS using a 660K single nucleotide polymorphism (SNP) array was conducted to identify significant QTLs and candidate genes for GZnC. Thus, this study aimed to provide useful information for further GZnC improvement for in wheat.

## 2 Materials and methods

### 2.1 Plant materials

Based on previous diversity assessments, the experimental material used herein comprised 382 representative wheat accessions, including 68 exotic cultivars, 43 landraces, and 271 domestic cultivars. These accessions were planted in the cropping season from 2017 to 2018 in Nanyang (33.03°N, 112.50°E), Suqian (34.02°N, 118.33°E), and Yangling (34.16°N, 108.40°E), where the soil Zn concentrations were 221.38, 384.86, and 233.25 mg/kg, respectively. The field was managed according to the local standard agronomic practices.

### 2.2 Determination of GZnC

Wheat grains were harvested manually in the field. Three biological replicates were sampled from each wheat accession in each environment. Wheat grains were washed with distilled water and dried to avoid potential contamination. Dried wheat grains were ground to whole wheat flour using a stainless steel grinder (MM400, Retsch, Haan, Germany), and whole wheat flour was subsequently oven-dried at 80 °C for 12 h. Subsequencely, 0.2 g of whole wheat flour was digested in 8 ml of a mixture of high-purity concentrated nitric acid and hydrogen peroxide (HNO_3_/H_2_O_2_, 75/25, v/v), and then diluted with ultrapure water. After filtration, the zinc concentration in the digestion solution was determined using an inductively coupled plasma system (iCAP 7000, Thermo Scientific) according to the method described by Ma et al. (2022). And a standard curve (0.07, 0.14, 0.21, 0.28, 0.35, 0.42 mg/L) was used for each round of determinations. Finally, the GZnC (mg/kg) was calculated on a dried weight basis.

### 2.3 Statistical analysis

Analysis of variance (ANOVA) and *t*-test were performed using SPSS 26.0. The following formula was used for estimating generalised heritability (*H* ^2^):


$$H^{2}=\frac{\sigma_{G}^{2}}{\sigma_{G}^{2}+\sigma_{GE}^{2}/n+\sigma_{e}^{2}/nr}$$

Where $\sigma_{G}^{2}$ represents the phenotypic variation due to the genotype, $\sigma_{GE}^{2}$ represents the phenotypic variation due to the environment × genotype interaction, $\sigma_{e}^{2}$ represents error variance, *n* represents the number of environments, and *r* represents the number of replicates. The best linear unbiased predictions (BLUPs) of GZnC for each accession across the three environments were calculated using the mixed linear model in the “lem4” package, version 3.5.3 of R, and were used for further GWAS.

### 2.4 SNP genotyping and screening

The Affymetrix Wheat 660K SNP array was used to genotype the wheat panel. SNP genotype calls and allele clustering were analyzed using the Affmetrix Genotyping Console software. The parameters of allele frequency (MAF) less than 0.05 and missing data greater than 10% were used to filter the SNP marker to ensure the accuracy of genotyping, together with a Hardy-Weinberg equilibrium value greater than 0.01 (Wu et al., 2021), resulting in a total of 405,606 high-quality SNP markers for GWAS. The physical locations of all SNP markers were determined using the bread wheat reference genome of IWGSC RefSeq v1.0 (I.W.G.S.C, 2018).

### 2.5 Linkage disequilibrium analysis, population structure and GWAS

Genome-wide LD analysis was performed using PLINK. The squared correlation (*r* ^2^) of allele frequencies was plotted in R Studio using genetic distance (Mb). The LD decay pattern was determined as the distance at which the LD value was reduced to half of its maximum value. STRUCTURE v2.3.4 (unlinked markers (*r* ^2^ = 0)) was used to calculate population structure (Pritchard et al., 2000). Each K value with a burn-in length of 20,000 was calculated five times, and iterations were set to be 10,000. The delta K (ΔK) method was used to determine the most likely number of subpopulations based on the change rate between the K values (Earl and vonHoldt, 2012). The general linear model in the GEMMA software was used to conduct GWAS, and BLUPs of 382 wheat accessions were used as phenotypic data for GWAS. After calculating the suggestive *P*-value threshold based on Bonferroni’s correction (*P* = 1/Ne, where Ne represents the effective number of independent SNPs), we considered -log10 (*P-*value) ≥ 4.5 to be significant. The -log10 (*P-*value) values for all SNPs were plotted on a Manhattan plot.

### 2.6 Candidate gene identification

Non-synonymous mutations were obtained using significant SNPs in the selected QTLs, and phenotypic differences between haplotypes in the three environments were analyzed to identify the candidate genes. Root, stem, and leaf tissues at the jointing and heading stages, as well as wheat grains at 5, 10, 20, and 25 days post-anthesis (DPA), were collected from cv. Chinese Spring, which has been used for genome sequencing (I.W.G.S.C, 2018), for quantitative real-time PCR (qRT-PCR). Total RNA of each sample was extracted using TRIzol reagent (TaKaRa, Japan) following the manufacturer instructions, and first-strand cDNA was synthesised uisng the HiScript III First-stand cDNA synthesis kit (Vazyme, Nanjing, China). In turn, qPCR was conducted on an ABI 7500 real-time PCR system (Applied Biosystems, USA) using a SYBR Premix Ex Taq Kit (TaKaRa, Japan). *TaTubulin* was used as an internal control, and the related expression levels were calculated using the 2^−△△Ct^ method. Primers for qRT-PCR are listed in **Table S1**.

## 3 Results

### 3.1 Phenotypic analysis of GZnC

**Figure 1:**
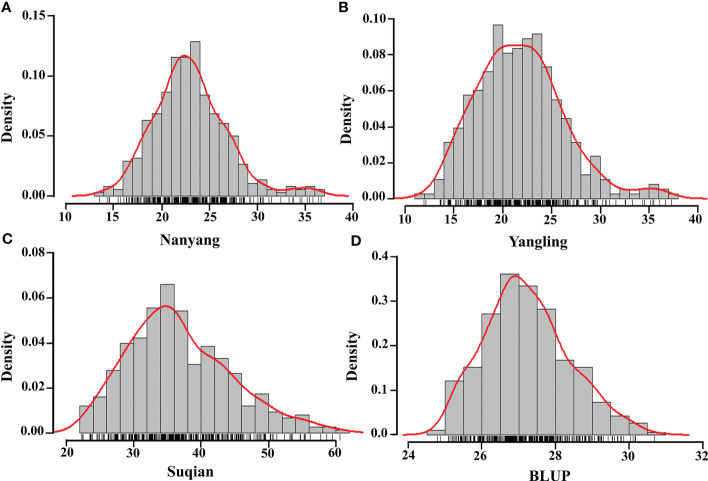
Histogram of wheat GZnC density in three environments and BLUP. Nanyang **(A)**, Yangling **(B)**, Suqian **(C)**, BLUP **(D)**, and the red curves and the black short horizontal lines represent the density curve and the rug plot of the distribution of GZnC.

The GZnC of 382 wheat accessions in three environments was determined (**Table S2**), and the corresponding maximum, minimum, median, variance, coefficient of variation, skewness, and kurtosis were calculated (**Table 1**). The highest GZnC values were recorded in Suqian, the environment with the highest soil Zn concentration, and ranged from 22.31 to 92.38 mg/kg (average 35.73 mg/kg). Those recorded in Nanyang followed, ranging from 13.55 to 60.59 mg/kg (average 22.64 mg/kg). In turn, the wheat accessions in Yangling showed lowest GZnC, ranging from 11.92 to 36.65 mg/kg (average 21.50 mg/kg). BLUPs of GZnC based on three environments were calculated in the range 24.78~39.21 mg/kg. ANOVA revealed that the genotype, environment, and interaction of genotype and environment had significant effects on GZnC (*P*<0.001). The *H* ^2^ of GZnC was determined to be 0.32, confirming that both environment and genotype had significant effects on GZnC (**Table S3**). In addition, the GZnC showed an approximately normal distribution in the three environments and BLUPs indicated by skewness, kurtosis, and GZnC distribution (**Table 1**; **Figure 1**). These findings indicated that the wheat panel was suitable for GWAS.

**Table 1:** Phenotypic data of GZnC in three environments and BLUP.

Environment	Min (mg/kg)	Max (mg/kg)	Mean (mg/kg)	Standard deviation (mg/kg)	Coefficient of variation (%)	Skewness	Kurtosis
Yangling	11.92	36.65	21.50	4.64	0.21	0.62	0.65
Nanyang	13.55	60.59	22.64	4.79	0.21	0.67	1.14
Suqian	22.31	92.38	35.73	9.41	0.26	0.57	0.02
BLUP	24.78	38.21	27.10	1.14	0.04	0.38	-0.20

### 3.2 GZnC variation with source and released years

**Figure 2:**
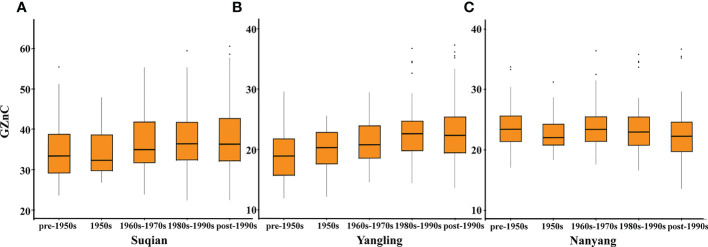
The changes of GZnC in Suqian **(A)**, Yangling **(B)**, and Nanyang **(C)** with released years.

Based on source, these wheat accessions were divided into three categories: 68 exotic cultivars, 43 landraces, and 271 domestic cultivars. Domestic cultivars showed the highest wheat GZnC, followed by landraces and exotic cultivars (**Table 2**). These wheat accessions were further divided into five stages according to the released years. In this respect, we found that GZnC increased from the pre-1950s to the post-1990s in Suqian and Yangling (**Figures 2A, B**), while it showed slightly fluctuation in Nanyang (**Figure 2C**). These findings indicate that the predominant allele may be selected during wheat breeding.

**Table 2:** GZnC of different source in three environments.

Wheat accessions	Nanyang (mg/kg)	Suqian (mg/kg)	Yangling (mg/kg)
Exotic cultivars	22.93	36.46	19.96
Landraces	23.42	34.56	20.03
Domestic cultivars	22.98	37.30	22.56

### 3.3 Genotyping by SNP array and linkage disequilibrium analysis

The 660K SNP array was used for conducting a GWAS using the phenotypes of 382 wheat accessions. After removing low-quality SNPs (MAF< 0.05 and missing data > 0.1), 412,619 SNPs remained for subsequent analysis. A set of 405,606 SNPs was distributed on 21 chromosomes, of which the B subgenome (185,057) was the most labelled, followed by the A subgenome (162,316), and the D genome (58,233). Chromosomal distribution of SNP markers showed that chromosome 3B contained the largest number of SNP markers (45,867) and the least number was found on chromosome 4D (4,046). Further, marker density distribution was uneven across chromosomes, ranging from 5.67 markers per Mb (4D) to 66.53 markers per Mb (3B). In addition, the polymorphism information content of the three sub-genomes was 0.28, 0.29, and 0.27, respectively (**Table 3**).

**Table 3:** Summary of the SNPs information in three sub-genomes and chromosomes of 382 wheat accessions.

Chromosome	No.of markers	Effective number	Effective Ratio	Suggestive *P*-value	Markers (%)	Length (Mb)	Marker density	He	PIC
1A	28,959	5,687	0.20	1.76E-04	7.14	594.02	48.75	0.66	0.27
1B	20,624	4,796	0.23	2.09E-04	5.08	780.76	26.42	0.76	0.30
1D	10,584	3,090	0.29	3.24E-04	2.61	750.73	14.10	0.67	0.28
2A	28,832	6,170	0.21	1.62E-04	7.11	744.54	38.72	0.70	0.28
2B	28,645	6,708	0.23	1.49E-04	7.06	709.76	40.36	0.73	0.29
2D	10,449	3,744	0.36	2.67E-04	2.58	617.97	16.91	0.69	0.28
3A	19,409	4,611	0.24	2.17E-04	4.79	736.69	26.35	0.69	0.28
3B	45,867	7,828	0.17	1.28E-04	11.31	689.38	66.53	0.77	0.31
3D	7,247	2,678	0.37	3.73E-04	1.79	801.25	9.04	0.67	0.27
4A	17,856	4,258	0.24	2.35E-04	4.40	830.70	21.50	0.67	0.27
4B	13,120	3,021	0.23	3.31E-04	3.23	673.47	19.48	0.71	0.29
4D	4,046	1,666	0.41	6.00E-04	1.00	713.02	5.67	0.66	0.27
5A	22,616	4,947	0.22	2.02E-04	5.58	720.95	31.37	0.78	0.31
5B	33,966	6,539	0.19	1.53E-04	8.37	750.61	45.25	0.80	0.31
5D	8,396	3,267	0.39	3.06E-04	2.07	495.44	16.95	0.65	0.26
6A	16,383	3,834	0.23	2.61E-04	4.04	651.81	25.13	0.73	0.29
6B	25,783	5,565	0.22	1.80E-04	6.36	615.48	41.89	0.69	0.28
6D	7,425	2,924	0.39	3.42E-04	1.83	509.85	14.56	0.65	0.26
7A	28,261	6,280	0.22	1.59E-04	6.97	566.04	49.93	0.69	0.28
7B	17,052	4,499	0.26	2.22E-04	4.20	473.56	36.01	0.72	0.29
7D	10,086	3,682	0.37	2.72E-04	2.49	638.65	15.79	0.64	0.26
A genome	162,316					4934.47	32.89	0.70	0.28
B genome	185,057					5719.38	32.36	0.74	0.29
D genome	58,233					3950.83	14.74	0.66	0.27
Total	405,606					14064.68	28.84	0.70	0.28
Average				2.51E-04					

**Figure 3:**
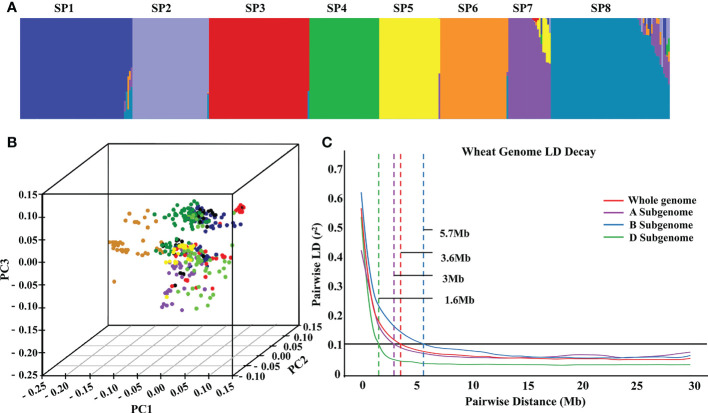
The population structure and linkage disequilibrium of 382 wheat accessions. Subpopulations inferred by K-mean structure analysis **(A)**. Principal component analysis of all wheat accessions **(B)**. LD decay **(C)** over different genetic distances (Mb) for A, B and D subgenomes and whole genome in the wheat panel (*r* ^2^ = 0.1).

Population structure analysis was performed for these 382 wheat accessions. According to the *Δ*K method of Bayesian clustering, the slope broke when K = 8. As a result, the 382 wheat accessions were divided into eight subpopulations, SP1-SP8 (**Table S2**; **Figures 3A, B**). SP1 contained eight exotic cultivars, two landraces, and 56 domestic cultivars, with an average GZnC of 27.36 mg/kg. SP2 contained four exotic cultivars, 35 landraces, and six domestic cultivars, with an average GZnC of 26.04 mg/kg. SP3 contained 59 domestic cultivars, with an average GZnC of 27.31 mg/kg. SP4 contained one exotic cultivars, two landraces, and 34 domestic cultivars, with an average GZnC of 27.29 mg/kg. SP5 contained two exotic cultivars and 38 domestic cultivars, with an average GZnC of 28.10 mg/kg. SP6 contained one exotic cultivar, one landraces, and 38 domestic cultivars, with an average GZnC of 27.90 mg/kg. SP7 contained 25 exotic cultivars, with an average GZnC of 26.46 mg/kg. SP8 contained 27 exotic cultivars, three landraces, and 40 domestic cultivars, with an average GZnC of 27.14 mg/kg (**Table S4**). We found that the GZnC fluctuated slightly among the eight subpopulations. LD was estimated using SNPs and squared allele frequency correlations (*r* ^2^) for subgenomes A (162,316), B (185,057), and D (58,233). As expected, LD decayed with increasing physical distance, and it differed among subgenomes. Further, whole-genome-wide LD decayed with genetic distance, and it decayed to half of the genome at a genetic distance of 3.6 Mb. Subgenomes A, B, and D were 3.0 Mb, 5.7 Mb, and 1.6 Mb, respectively (**Figure 3C**).

### 3.4 GWAS for GZnC

**Figure 4:**
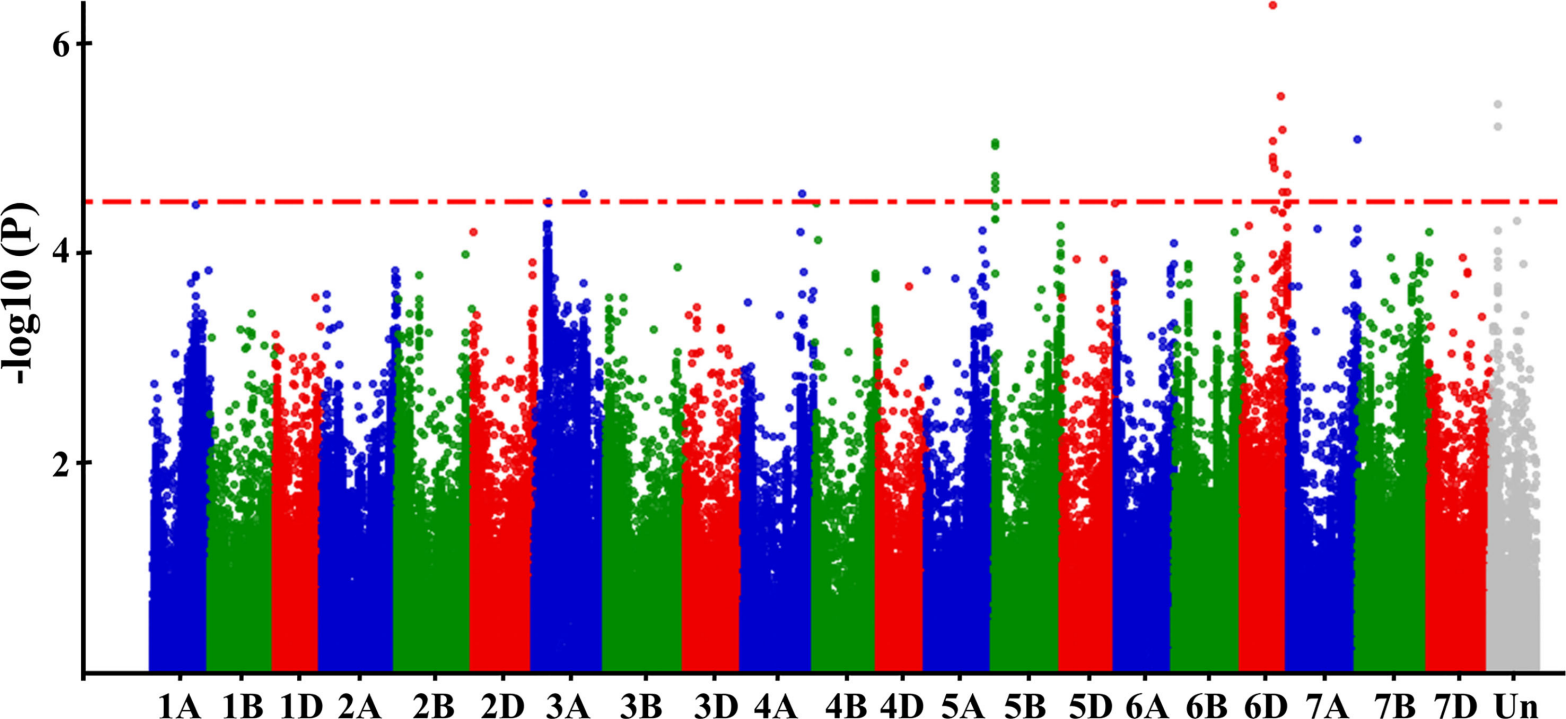
Manhattan plots of the GWAS results for GZnC. The resulting BLUPs of GZnC of 382 wheat accessions across three environments was used for GWAS. -log10 (*P*-value) of each SNP was shown in the Manhattan plot.

The BLUPs based on the GZnC across three environments were used for the GWAS using the Lm4 model. SNPs with –log10 (*P*-value) ≥ 4.5 were deemed significant (**Figure 4**). A total of nine significant QTLs were selected, locating on chromosomes 3A, 4A, 5B, 6D, and 7A. For convenience, the SNP markers with the highest threshold in each QTL were used to represent the corresponding QTL, and the effects of the nine QTLs ranged from 0.16 to 0.30 (**Table 4**).

**Table 4:** Significant QTLs associated with GZnC.

Trait^a^	Chromosome	Marker^b^	Physical position^c^ (bp)	Predominant allele and its ratio^d^	-log_10_(*P-value*)	Effect
GZnC	3A	AX-109368604	140,810,282	G/A (0.12)	4.50	0.19
	4A	AX-109872817	621,769,441	G/A (0.38)	4.57	0.20
	5B	AX-109350479	13,177,703	G/T (0.46)	5.06	0.21
	6D	AX-108814900	307,646,180	G/A (0.39)	6.38	0.30
	6D	AX-108884748	328,977,424	A/C (0.33)	4.81	0.26
	6D	AX-89576368	403,654,827	T/C (0.45)	5.51	0.27
	6D	AX-108812106	410,167,022	C/T (0.49)	5.19	0.16
	6D	AX-94571657	469,831,635	C/G (0.11)	4.76	0.29
	7A	AX-109999591	726,436,979	T/C (0.33)	5.08	0.29

a GZnC, grain zinc concentration.

b The SNP markers with the highest threshold in each QTL were used to represent the corresponding QTL.

c Physical positions of single nucleotide polymorphism (SNP) markers were based on IWGSC RefSeq v.1.0.

d “–“ indicates the predominant allele with its ratio on GZnC.

### 3.5 Identification of candidate genes for wheat GZnC

A total of 494 genes were found in the nien QTLs. The functional annotations of these genes are provided in **Table S5**. We further analyzed significant SNPs in the nine QTLs that might cause missense mutations. Interestingly, we found that the phenotype between alleles, which was caused by the SNP AX-108884748, showed significant differences in the three environments, whereby it was further analyzed, as it may be closely related to GZnC.

**Figure 5:**
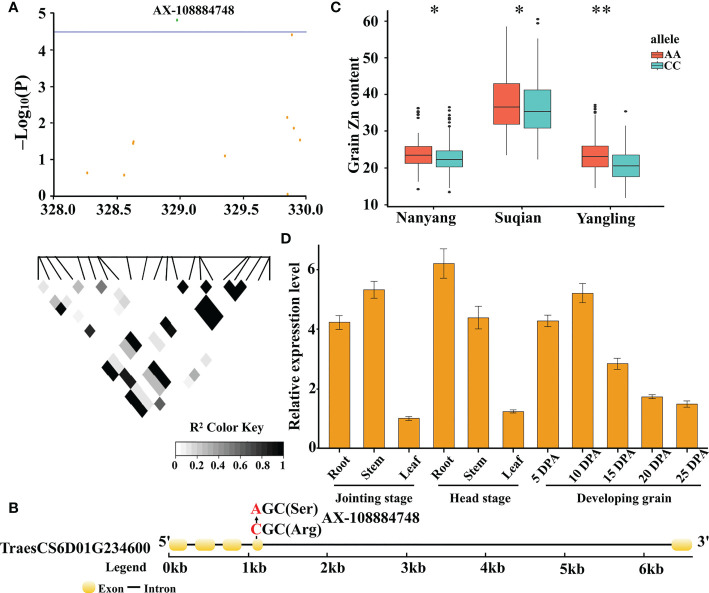
Candidate gene analysis based on the SNP AX-108884748 on chromosome 6D. Local Manhattan plot for the candidate region on chromosome 6D. The green dot represents the significant SNP AX-108884748. The corresponding LD block analysis of SNPs in this region is shown below. The degree of linkage is represented by the coefficient of *r* ^2^ **(A)**. Gene structure and location of the non-synonymous SNP for *TraesCS6D01G234600*. Yellow rectangles and black lines represent exons and introns, respectively **(B)**. Boxplots for GZnC based on haplotype analysis **(C)**. Upper and lower edges of the box represent the 75th and 25th quantiles, respectively, and the whiskers show the 90th and 10th quantiles; the horizontal solid lines represent the median. Statistical significance was analyzed by *t*-test, * *P* ≤ 0.05; ** *P* ≤ 0.01. Expression profile of *TraesCS6D01G234600* in different tissues, as determined by qRT-PCR. The data was shown as the means ± Sd of *n* = 3 **(D)**.

The SNP, AX-108884748, is located at 328,977,424 bp on chromosome 6D (**Figure 5A**), and may cause an amino acid change from Arg (CC haplotype) to Ser (AA haplotype) at 1047 bp in the GDSL esterase-encoding gene, *TraesCS6D01G234600* (**Figure 5B**). The 382 wheat accessions were divided into three categories according to genotype: among them, 250 wheat accessions with the CC haplotype, 123 with the AA haplotype, and 9 with heterozygosity or lack of genotype. After analysing the phenotypic data of the two haplotypes, the GZnC of the AA haplotype was found to be significantly higher (*P* ≤ 0.05) than that of the CC haplotype across three environments (**Figure 5C**). Further, qRT-PCR analysis showed that the gene was predominantly expressed in the root, stem, and grain tissues, suggesting a possible role in Zn transfer (**Figure 5D**). Altogether, these results indicate that *TraesCS6D01G234600* is an important candidate gene for wheat GZnC.

## 4 Discussion

### 4.1 Increasing wheat GZnC is needed to alleviate “hidden hunger”

Wheat grain yields have significantly increased over the past 75 years, from the early days of the “Green Revolution” in the 1960s to the recent optimisation of breeding and agronomic crop management (Evenson and Gollin, 2003; Pingali, 2012). However, nutrition-related traits are often neglected in the breeding process, resulting in the low concentration of microelements in wheat grains, giving rise to what has been called “hidden hunger” (Ul-Allah, 2018). Zn is an indispensable microelement for the human body. As an essential microelement, Zn cannot be synthesised by the human body, and plays a very important role in the human nervous, immune, and reproductive systems, as well as in the growth and development of children. Hao et al. analyzed wheat GZnC over the past 80 years in China, and found no decrease with released years (Hao et al., 2021). In turn, this study revealed that wheat GZnC has increased with the released years. Therefore, the dominant allele of wheat GZnC was never lost during selective breeding.

As one of the main food crops in the world, bread wheat is the staple food and major source of microelements for 30% to 40% of the world population (Poudel and Bhatta, 2017). Given the significant impact of Zn on human health (Shewry et al., 2012), it is unfortunate that, as many studies have shown, wheat GZnC is low worldwide. Thus, for example, Rehman et al. analyzed 28 wheat accessions in Pakistan and found that wheat GZnC ranged from 21.20 to 54.40 mg/kg (Rehman et al., 2018). Similarly, Khokhar et al. analyzed 245 wheat accessions from Woestkin and found that GZnC ranged from 24.0 to 49.0 mg/kg (Khokhar et al., 2020). In turn, Maryami et al. analyzed 158 Iranian wheat accessions and found that GZnC ranged from 27.9 to 65.0 mg/kg (Maryami et al., 2020). In particular, according to a summary and analysis of a large number of studies worldwide by Wang et al., the average global zinc concentration in wheat grains is only 28.48 mg/kg (Wang et al., 2020). Consistently, the results reported herein revealed that the GZnC ranged from 11.92 to 92.83 mg/kg. Although domestic wheat cultivars contained the highest GZnC, they were still far below human requirements, at approximately 40 to 60 mg/kg (Cakmak, 2008). Therefore, there is an urgent need to improve GZnC in wheat.

### 4.2 Genetic structure analysis was performed by comparing known loci

As a complementary strategy, GWAS is a powerful tool to for detecting QTLs for complex traits (Hamblin et al., 2011). With the development of wheat genome sequencing, different SNP arrays have been developed and gradually become the main tools for wheat GWAS (Rasheed et al., 2017). To reveal the QTLs for wheat GZnC, a 35K SNP array was used to identify two QTLs located on chromosomes 1A and 7B (Rathan et al., 2022). A wheat 660K SNP array was used to map seven QTLs on chromosomes 1B, 3B, 3D, 4A, 5A, 5B, and 7A (Zhou et al., 2020). Subsequently, the 90K and 660K SNP arrays were used to identify 17 QTLs on chromosomes 1A, 2A, 3A, 3B, 5A, 5D, 6A, 6B, 6D, 7A, 7B, and 7D, which were related to wheat GZnC (Tong et al., 2022). However, the molecular mechanism underlying Zn accumulation in wheat grains remains unclear, and no molecular marker conducive to GZnC has been used for wheat breeding. In this study, we identified nine QTLs on chromosomes 3A, 4A, 5B, 6D, and 7A. Specifically, the QTL (AX-108884748) was first identified on chromosome 6D, and is most likely a novel QTL located in the chromosomal region. This finding enriches our understanding of the genetic basis of GZnC in wheat.

### 4.3 Candidate genes for GZnC in wheat

A few genes have been functionally verified aiming to improve wheat GZnC. For example, the NAC transcription factor, NAM-B1, promotes the transfer of Zn from the leaves to the grains to increase GZnC (Uauy et al., 2006). However, wheat GZnC is a quantitative trait that is contributed by many genes. To date, many studies have been performed using GWAS to screen candidate genes for complex traits in wheat. Tong et al. used three methods: haplotype analysis, gene function comparison, and lineal homologues to screen a total of 28 promising candidate genes that might be involved in zinc/iron absorption, transport, storage, and regulation (Tong et al., 2022). In turn, Zhou et al. used superior allele estimation to identify seven candidate genes for GZnC, encoding NAC transcription factor and TPR-like superfamily proteins (Zhou et al., 2020). Meanwhile, using haplotype analysis, Krishnappa et al. identified two important candidate genes for GZnC, encoding a late embryogenic rich protein, *LEA-18*, and RNA recognition motif domains (Krishnappa et al., 2022). These findings indicate that GWAS is a highly useful tool for identifying candidate genes for complex traits. In addition, these studies showed haplotype analysis is reliable for screening candidate genes for complex traits, which laid a solid theoretical foundation for our subsequent research.

In this study, we identified the candidate gene *TraesCS6D01G234600* on chromosome 6D through haplotype analysis, which was caused by the SNP AX-108884748 (CC/AA). This candidate gene encodes GDSL esterase, which shows hydrolytic enzyme activity for thioesters, aryl esters, phospholipids and amino acids, and others (Akoh et al., 2004). The AA allele had a significant positive effect on GZnC in all three environments (*P* ≤ 0.05), indicating the importance of *TraesCS6D01G234600*. In addition, this gene was mainly expressed in the root, stem, and grain tissues, suggesting that it may play an important role in Zn transfer. These results provide important information for further improvement of wheat GZnC, and indicate that the pyramid effect should be considered in the next step of selective breeding.

## 5 Conclusion

We constructed a diversity panel comprising 382 wheat accessions, and conducted field trials in three environments. The GZnC of 382 wheat accessions was determined, and the data showed that wheat GZnC increased with the release years, indicating that the predominant allele of wheat GZnC was not lost during selected breeding. Phenotypic data were further analyzed using GWAS, and nine QTLs for GZnC were identified, with effects ranging from 0.16 to 0.30. In addition, one candidate gene for GZnC was screened using haplotype analysis. Overall, our study provides novel insights that increase our understanding of the genetic information of GZnC and will facilitate the improvement of GZnC in wheat breeding programs.

## Data availability statement

The original contributions presented in the study are included in the article/**Supplementary Material**. Further inquiries can be directed to the corresponding authors.

## Author contributions

JM, DH, and LJ conceived and designed the research. JM, MYe, QL, MYuan, DZ, CL, QZ, and JW performed the experiments. JM, MYe, and MYuan prepared the figures and provided the materials. JM wrote the manuscript. All authors have read and approved the final manuscript.
